# Morphogenesis of the carapace from phyllosoma to puerulus in the Japanese spiny lobster

**DOI:** 10.1186/s40851-026-00265-8

**Published:** 2026-04-02

**Authors:** Haruhiko Adachi, Kentaro Morikawa, Yasuhiro Inoue, Shigeru Kondo

**Affiliations:** 1 Institute for Advanced Biosciences, Keio University, Tsuruoka, Yamagata 997- 0017 Japan; 2 Graduate School of Media and Governance, Keio University, Fujisawa, Kanagawa 252-0882 Japan; 3 Department of Micro Engineering, Graduate School of Engineering, Kyoto University, Kyoto, 615-8540 Japan; 4 National Institute of Genetics, Mishima, Shizuoka 411-8540 Japan

**Keywords:** Phyllosoma, Puerulus, Metamorphosis, Morphogenesis, Molting

## Abstract

Molting in exoskeleton-bearing organisms can result in dramatic body shape transformations. While this phenomenon has been well-studied in insects, limited knowledge exists for other animals, including crustaceans. Among crustaceans, achelate lobsters, such as Palinuridae and Scyllaridae, exhibit a unique metamorphic transition from a flattened phyllosoma larva to a three-dimensional puerulus or nisto stage in a single molt. This study investigates the mechanisms underlying this transformation in the Japanese spiny lobster (*Panulirus japonicus*), focusing on carapace deformation through histological analysis, live imaging, and computational modeling. Three-dimensional micro-computed tomography revealed a significant reduction in body length and a shift in mouth position during metamorphosis. Live imaging captured dynamic morphological changes, including epithelial contraction and subsequent reshaping. Furthermore, observation of the transient metamorphosis phase identified a distinctive furrow structure on the carapace surface in a mid-metamorphosis specimen that died during the metamorphosis process, absent in post-metamorphosis puerulus larvae, suggesting a possible role in the transformation process. To explore the formation of this furrow pattern, a computational model was developed based on differential shrinkage rates in vertical and horizontal directions. The model successfully reproduced similar patterns observed in natural specimens, implying that controlled anisotropic contraction could contribute to morphogenesis. Furthermore, three-dimensional shrinkage simulations demonstrated that local contraction with constrained out-of-plane deformation can generate folds, which later expand to form curved structures. This study provides novel insights into the biomechanics of arthropod molting, highlighting a previously unrecognized mechanism of two-dimensional-to-three-dimensional transformation. The findings enhance our understanding of larval development in Achelata and offer broader implications for exoskeletal morphogenesis across arthropods.

**Supplementary Information:**

The online version contains supplementary material available at 10.1186/s40851-026-00265-8.

## Background

In animals with exoskeletons, molting can cause extreme changes in body shape. The mechanism has been studied in detail in insects, including flies [1] and beetles [2, 3], but little is known about other taxa. Crustaceans also exhibit diverse larval morphologies depending on the species and undergo drastic morphological changes through the process of molting [4]. Among crustaceans, the infraorder Achelata possesses a characteristic developmental style. Members of the families Palinuridae and Scyllaridae begin their life cycle with a very flat morphology (Mov. 1), called phyllosoma larvae, although their outlines differ [5]. After repeated molting, the final instar phyllosoma larvae undergo metamorphosis in which they transform into puerulus larvae in Palinuridae and nisto larvae in Scyllaridae with a single molt, and then molt into the juvenile phase [6, 7]. In this study, we distinguish between these terms: “metamorphosis” refers to the drastic rearrangement of the body plan from the planktonic phyllosoma to the nektonic puerulus, while “molting” refers to the specific ecdysis event that facilitates this transformation. A critical structural difference lies in the cephalothorax. In phyllosoma larvae, the cephalic and thoracic regions are arranged in a flattened plane covered by a cephalothoracic shield, lacking a fully developed dorsal carapace. In contrast, the puerulus stage is characterized by the integration of the head and thorax into a single, curved three-dimensional structure known as the carapace. Thus, the transition from a flat, two-dimensional form to a three-dimensional form is physically executed during a single molting event. In order to elucidate the mechanism of transformation from a flat to a three-dimensional state, we focused our attention on the morphogenesis of the Japanese spiny lobster.

With regard to the phyllosoma to puerulus metamorphosis, temporal transcriptome analysis of *Sagmariasus verreauxi* and *Panulirus ornatus* has been conducted, and sequences that are characteristically expressed at each stage have been found [8, 9]. The cross-sectional structure of the cuticle at each stage has also been analyzed, and the oriented chitin fiber structure seen in general crustaceans has not been observed in phyllosoma larvae, and a characteristic amorphous calcium carbonate structure has been confirmed. Then, although calcium carbonate usually has a crystalline structure, proteins that are thought to be important for maintaining an amorphous state in phyllosoma have also been identified [10]. In addition, changes in the elemental composition during the development of phyllosoma have recently been captured [11]. Aquaculture studies have also been conducted in Japan for a long time in species such as *Panulirus japonicus* and *Panulirus penicillatus*, and the developmental process including the larval stage has been described [7, 12, 13], as well as the observation of the dynamics during metamorphosis [14]. On the other hand, the curvature of the carapace, which is characteristic of the transformation from phyllosoma to puerulus, remains poorly understood. In this study, we focused on the deformation of the carapace, analyzed what kind of deformation is occurring using histological analysis and live observation at the time of metamorphosis, and proposed a model of the deformation.

## Methods

### Animals

***Panulirus*** *japonicus* lobsters were cultured at the Mie Prefectural Science and Technology Promotion Center, Fisheries Research Division. The culture conditions were identical to those employed in previous studies [13]. The specimens were fixed in 70% ethanol. For the quantitative morphological analysis of body shape changes, we used three final instar phyllosoma larvae (pre-metamorphosis stage) and three puerulus larvae (post-metamorphosis stage). To observe the transient morphological features during the metamorphic process, we examined one specimen that died during the metamorphosis process. Metamorphosis behavior was recorded using two video cameras: an FDR-AX (Sony, Japan) and an iPhone SE2 (Apple, USA). Metamorphosis onset was determined by antenna folding, following a previous study [14].

### Micro-CT (µCT)

Following established methods in insect studies [15], specimens fixed in 70% ethanol were transferred to 100% ethanol and finally to *t-*BtOH. Specimens were then frozen at 4 °C and dried using a freeze dryer to minimize deformation. Dried specimens were scanned using a µCT Skyscan 1172 (Bruker, Belgium) and reconstructed with NRecon ver. 1.7.0. Mesh files were also output as STL data for later three-dimensional simulations. Cross-sectional images were obtained using CTAn. µCT allowed us to accurately measure the arc length along the curved carapace, which is impossible to assess with conventional two-dimensional microscopy. We examined phyllosoma larvae (*n* = 3), puerulus larvae (*n* = 3), and the mid-metamorphosis specimen that died during the metamorphosis process (*n* = 1). Morphological measurements were performed using Fiji [16]. Specifically, we measured the body length, the shift in mouth position, and the “dorsal side length,” which is defined as the arc length of the dorsal carapace. (*n* = 3 for each stage)

### Scanning electron microscopy (SEM)

CT-scanned specimens were sputter-coated with gold and used for SEM analysis. They were attached to carbon seals and imaged at 10 kV on a JCM-6000 NeoScope (JEOL, Japan). The resulting images were concatenated using the Fiji plugin Image Stitching [17]. The furrow structures on the carapace surface were examined in detail using the mid-metamorphosis specimen. For cross-sectional observation of the cuticle, the samples used for µCT and SEM were embedded in UV resin. These samples were sectioned at 50 μm using a microtome, mounted on conductive tape, and observed (*n* = 1 for each stage).

### Expansion simulation

This followed methods established for beetle horns [3, 18]. Calculations were performed to flatten the angle between adjacent triangular meshes while preserving their area. Implementation was carried out in Julia language ver 1.8.

### Two-dimensional computational shrinkage

The toy model was performed in Julia language ver. 1.8. Displacements of ellipses and rectangles were placed on each side of the rectangle and distributed inside the rectangle to simulate a quasi-contraction (Fig. S3). The distribution was carried out according to a Gaussian distribution, in which the internal influence is varied by the variance parameter σ. Protruding ends were distributed vertically and horizontally based on the aspect ratio during the initial arrangement and internally according to a Gaussian distribution. When σ is small, the pattern stops on the perimeter, and when σ is large, it penetrates more inside.

### Three-dimensional computational shrinkage

We represented the carapace epithelium as a triangular mesh and simulated its deformation by computing the motion of mesh vertices based on a mechanical model. The motion of each vertex was assumed to obey an overdamped (viscous-dominated) equation of motion, and the force acting on each vertex was given by the gradient of the total energy, which consisted of stretching, bending, and $z$-directional displacement-constraint energy terms [19]. The $z$-directional displacement-constraint energy represents the restriction of out-of-plane deformation imposed by the cuticle. This model was used both for simulations of furrow formation induced by shrinkage and for simulations of furrow unfolding.

### Equation of motion for vertex 𝒊 of a triangular mesh

Assuming viscous-dominated dynamics, the equation of motion for vertex $i$ with position ${x}_{i}$ is written as$\gamma \frac{dx_i}{dt}=-\frac{\partial E}{\partial xi}$.

$$E=E^{stretch}+E^{bend}+E^z$$

where $\gamma$ is a friction coefficient and $E$ is the total energy. In all simulations, we set $\gamma=1.0$.

### Details of each energy function

#### Energy of stretching [20]

The in-plane strain ${\varepsilon}_{i}$ due to stretching in the triangular mesh was expressed, for each triangle $i$, using its discrete first fundamental form ${I}_{i}$ as follows:

$${\varepsilon}_{i}:=(I_i^{tgt} )^{-1} (I_i-I_i^{tgt} )$$ 

$$I_i := \left(\begin{array}{cc}\|e_{i_1}\|^2 & e_{i_1}\cdot e_{i_2} \\e_{i_1}\cdot e_{i_2} & \|e_{i_1}\|^2\end{array}\right)$$

where ${I}_{i}^\mathrm{tgt}$ is the first fundamental form of triangle $i$ in the reference (target) configuration and ${e}_{{i}_{j}}$ denotes the vector of edge $j$ of triangle $i$. The stretching energy was written using material parameters $\alpha=\frac{E\nu}{1-{\nu}^{2}},\beta=\frac{E}{2\left(1+\nu\right)}$ where $E$ is the Young’s modulus and $\nu$ is the Poisson’s ratio:

$$E^{\mathrm{stretch}}=\sum_{i}^{\mathrm{face}}\frac{h}{4}\frac{\sqrt{\det I_i^{\mathrm{tgt}}}}{2}\left(\frac{\alpha}{2}\,\mathrm{tr}^2 \varepsilon_i+\beta\,\mathrm{tr}(\varepsilon_i^2)\right)$$ 

where $h$ denotes the thickness. In all simulations, we set $\alpha=6.6\times{10}^{3},\beta=7.7\times{10}^{3},h=1.0\times{10}^{-2}$. In the shrinkage simulations, to realize anisotropic shrinkage, we set ${I}_{i}^\mathrm{tgt}$ as the shape obtained from the initial shape ${I}_{i}^\mathrm{init}$ by scaling it by a factor ${r}^{x}=\frac{2}{3}$ in the $x$-direction (the major axis of the initial elliptical shape) and by a factor ${r}_{i}^\mathrm{A}$ in area. The target area was thus written as ${A}_{i}^\mathrm{tgt}={r}_{i}^\mathrm{A}{A}_{i}^\mathrm{init}$; the procedure for specifying ${A}_{i}^{tgt}$ is described below.

#### Energy of bending [21]

As the bending energy of the triangular mesh, we adopted an energy function based on the dihedral angle ${\theta}_{i}$ between the two triangles sharing each edge $i$:

$$E^{\mathrm{bend}}=\sum_{i}^{\mathrm{edge}}k_\mathrm{DA}\frac{3 (L_i^{\mathrm{tgt}})^2}{A_{i_1}^{\mathrm{tgt}} + A_{i_2}^{\mathrm{tgt}}}\left( 2 \tan \frac{\theta_i}{2} \right)^2$$ 

where ${k}_\mathrm{DA}$ is an elastic coefficient, ${L}_{i}^\mathrm{tgt}$ is the length of the edge $i$ in the reference configuration, and ${A}_{{i}_{1}}^\mathrm{tgt},{A}_{{i}_{2}}^\mathrm{tgt}$ are the areas of triangles sharing the edge $i$ in the reference configuration. In the shrinkage simulations, this term ${E}^\mathrm{bend}$ was interpreted as the mechanical bending energy of the material. In the unfolding simulations, the same functional form was used as a geometric flattening energy to obtain the unfolded shape. We set ${k}_\mathrm{DA}=1.0\times{10}^{-4}$ in the shrinkage simulations and ${k}_\mathrm{DA}=1.0\times{10}^{-2}$ in the unfolding simulations.    

#### Energy of 𝒛-directional displacement constraint [19]

In the shrinkage simulations, the initial configuration of the tissue was taken to be an elliptical domain in the $xy$-plane, and we assumed that the cuticle constrains deformation in the $z$-direction. Accordingly, the $z$-directional displacement-constraint energy was defined as

$$E^{z}=\sum_{i}^{\mathrm{vertex}}\frac{1}{2} k_z z_i^2$$ 

where ${z}_{i}$ is the $z$-coordinate of vertex $i$. In the shrinkage simulations, we set ${k}_{z}=0.3$. In the unfolding simulations, where the cuticle constraint is absent, we set ${k}_{z}=0.0$.

#### Set-up formula for shrinkage rate distribution

In the shrinkage simulations, the target area ${A}_{i}^\mathrm{tgt}$ of each triangle $i$was defined relative to its initial area ${A}_{i}^\mathrm{init}$ as

$$A_i^{\mathrm{tgt}}=A_i^{\mathrm{init}}\left[1+a\left(1-\frac{1}{2\pi c^2}\exp\!\left(-\frac{\|x_i-b\|^2}{2c^2}\right)\right)\right]$$ 

where ${x}_{i}$ is the position of triangle $i$ (e.g. its centroid). This defines a shrinkage-rate distribution in which the target area reduction gradually increases with distance from the position $b$. The steepness of this gradient is controlled by the parameter $c$. In the simulations, we set $b=(1.1,0.0)$ and $c=0.5$. The parameter $a$ determines the overall magnitude of shrinkage. Guided by experimental observations, we chose $a$ so that the total area changes in the simulations matched the experimentally observed total area change, i.e. such that ${r}^\mathrm{total}=\frac{\sum_{i}{A}_{i}^\mathrm{tgt}}{\sum_{i}{A}_{i}^\mathrm{init}}=0.4$.

## Results and discussion

### Deformations during metamorphosis

**Fig. 1:**
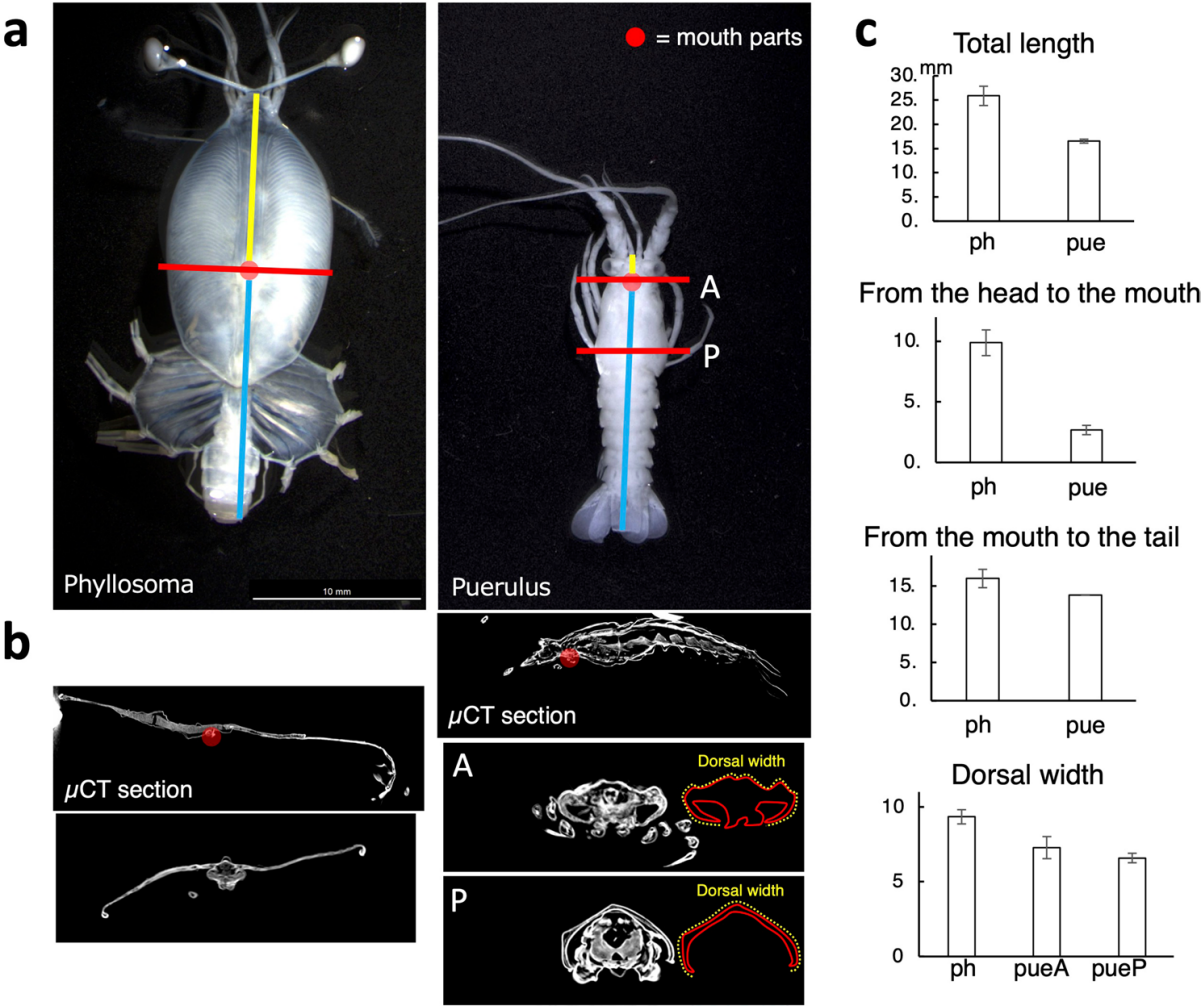
Morphology of phyllosoma and puerulus. (**a**) Bright-field images of phyllosoma and puerulus larvae. (**b**) Cross-sectional images using µCT, taken at the midline (sagittal plane; yellow and blue lines in (**a**)), at the mouth organ (red points in (**a**)), and at the widest point of the carapace (transverse plane; red line P in (**a**)). (**c**) Quantification of phyllosoma and puerulus morphology. Measurements include the ventral body length at the midline section, and the dorsal arc length of the transverse section (*n* = 3)

First, in order to ascertain the nature of the occurring deformations, the shape of the final instar phyllosoma and puerulus larvae was examined (Fig. 1a). Stereomicroscopic images do not allow observation of deformation in the depth direction; therefore, the three-dimensional morphology was analyzed using µCT (Fig. 1b). By analyzing the length from the top of the head to the tail and the length divided at the mouthpart from the cross-sectional view of the midline, the lengths from the top of the head to the mouth, from the mouth to the tail, and the total length were determined (Fig. 1c). The length from the head to the tail decreased by 72.9% (from 9.9 ± 1.1 mm to 2.7 ± 0.4 mm; mean ± SEM, *n* = 3) from phyllosoma to puerulus (Fig. 1c). Similarly, the length from mouth to tail decreased by 13.4% (from 16.0 ± 1.2 mm to 13.8 ± 0.02 mm) and the total length decreased by 36.1% (from 25.9 ± 2.0 mm to 16.5 ± 0.4 mm). Furthermore, the dorsal arc length was analyzed in the transverse direction using cross-sectional views. We measured the arc length at the mouthpart in the phyllosoma (9.34 ± 0.47 mm), and at both the mouthpart and the posterior widest part of the carapace in the puerulus. In the phyllosoma, the posterior arc length was not quantified due to the lack of distinct landmarks; however, visual inspection clearly confirms that the width decreases drastically toward the posterior end. The analysis confirmed that contraction occurred during metamorphosis. Following this contraction, the dorsal arc lengths at the mouthpart and the posterior region in the puerulus became comparable (7.28 ± 0.74 mm vs. 6.58 ± 0.32 mm; difference < 10%). This quantitative similarity supports the observation that the carapace morphology underwent a drastic transition from an elliptical shape to a sub-rectangular form, characterized by nearly parallel lateral margins. (Fig. 1c). Unlike previous studies limited to two-dimensional microscopy, our three-dimensional analysis using µCT enabled the accurate measurement of the arc length along the curved carapace—a parameter impossible to assess with conventional two-dimensional methods.

**Fig. 2:**
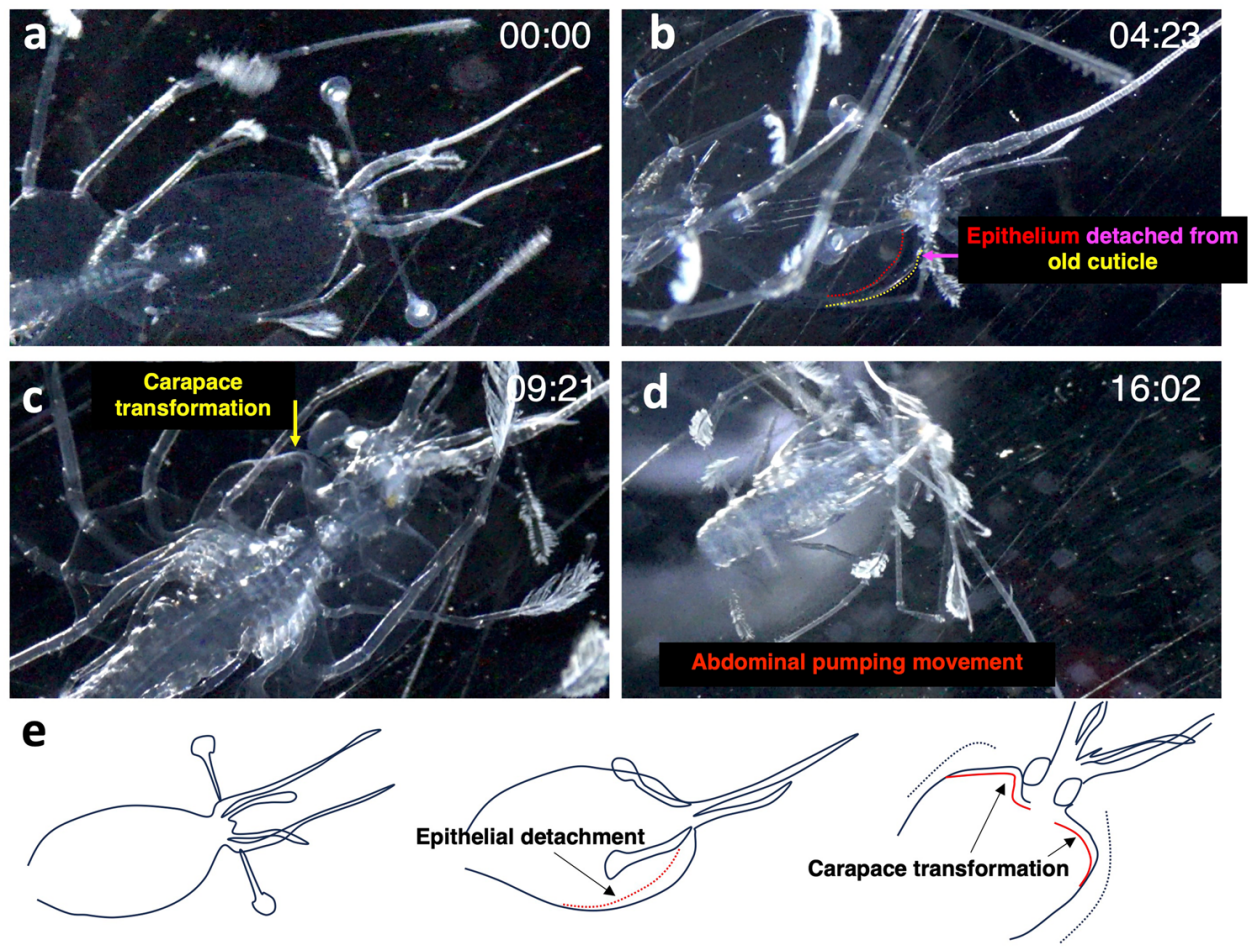
Live observation of the metamorphosis from phyllosoma to puerulus. (**a**) Early onset of metamorphosis. The ocular peduncles, a signal for metamorphosis onset, are beginning to collapse. No significant changes are observed in the carapace. (**b**) 4 min 23 sec after (**a**). The carapace epithelium can be seen detaching from the old cuticle. Yellow dotted line indicates old cuticle, red dotted line indicates new epidermis. (**c**) 9 min 21 sec after (**a**). The carapace undergoes contraction to transform locally into a three-dimensional structure. (**d**) 16 min 2 sec after (**a**). Pumping movements of the abdomen are observed. (**e**) Schematic representation of the carapace transformation process shown in (**a –c**)

In order to investigate the deformation on a shorter timescale, a video was taken at the time of metamorphosis (Fig. 2, Mov. 2). The entire process was successfully captured, beginning with the onset of the ocular peduncle falling and concluding with the puerulus form, which serves as a cue for the start of metamorphosis as previously observed in studies [14]. First, it was observed that the metamorphosis process, which lasts approximately 20 min, involves deformation from a planar structure to a shrimp-like form. Focusing on carapace deformation, we confirmed that the epithelial tissue first detached from the old cuticle and contracted (Fig. 2a, b,e, Mov. 3). Previous studies have demonstrated that gut retraction requires 3–4 days to reach completion in the cold-water species *Sagmariasus verreauxi* [8]. Although metabolic rates differ between species, the significant discrepancy between this multi-day timescale and the rapid event (approximately 20 min) observed in *Panulirus japonicus* suggests that the latter is driven by epithelial contraction. Subsequently, it was confirmed that the epithelial tissue contracted and locally transformed from a flattened to a three-dimensional form (Fig. 2c, e). Then, we observed that the puerulus form developed following a pumping movement of the abdomen (Fig. 2d, Mov. 4). Previous studies have observed the movement of organs during metamorphosis [14], but this study is the first to focus on the deformation of the carapace.

**Fig. 3:**
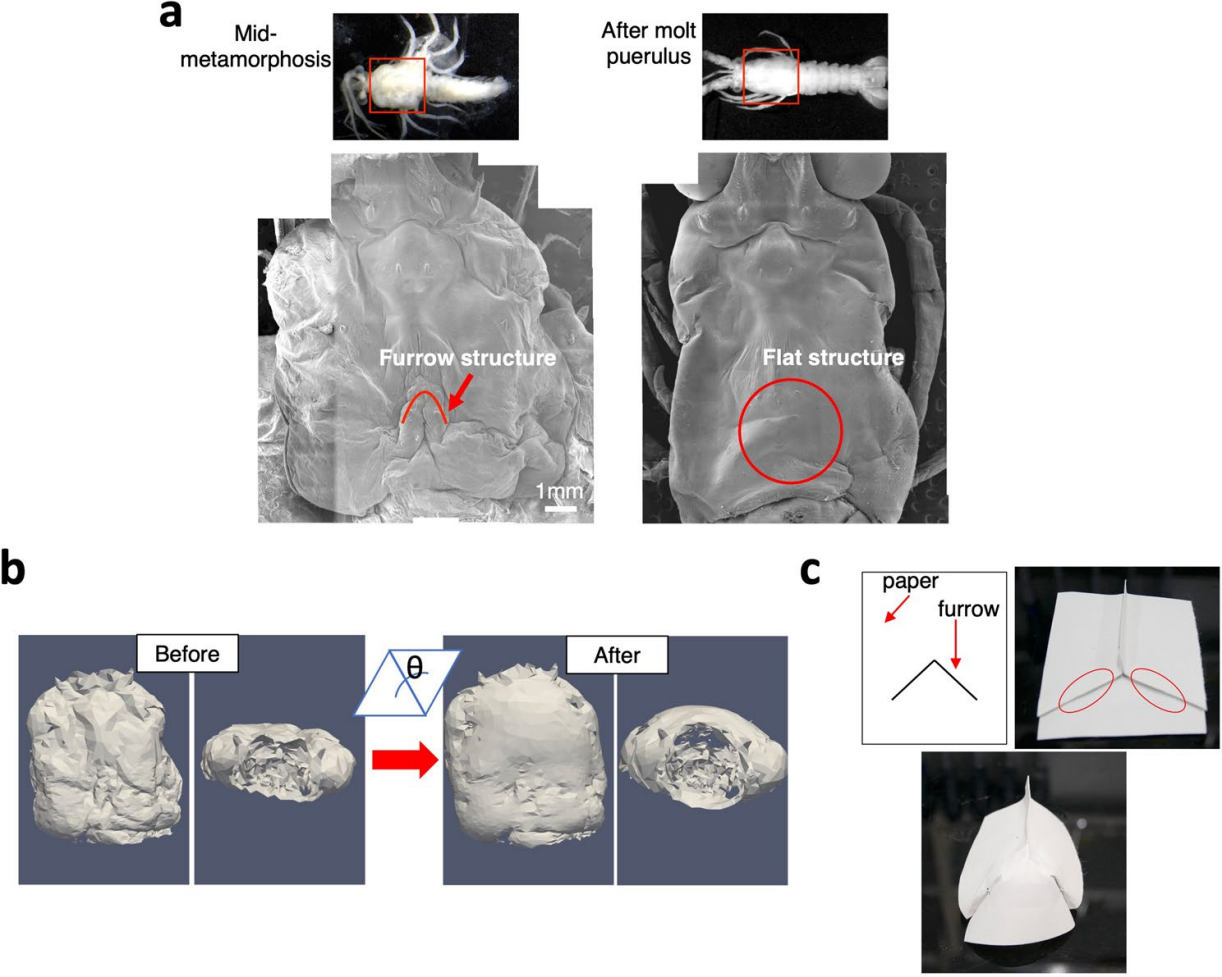
Transformation of the cuticular surface structure and simulation of furrow extension. (**a**) Cuticular surface of a mid-metamorphosis specimen that died during the metamorphosis process and post-metamorphosis puerulus. Distinctive furrow structures are observed on the mid-metamorphosis specimen, but are absent in the post-metamorphosis individuals. (**b**) Computational simulation of furrow extension on the mid-metamorphosis carapace reconstructed using µCT. The simulation demonstrates global carapace curvature resulting from furrow extension. (**c**) Reproduction of furrow patterns on paper and their unfolding process. Unfolding the paper model recreates the global curvature

We opportunistically examined a single specimen that died during the metamorphosis process. Bright-field observation confirmed that the individual remained partially encased in the old cuticle (Fig. 3a, Fig. S1), indicating death prior to complete exuviation. After carefully removing the old cuticle, SEM analysis revealed the presence of developing chitin fibers on the surface of the contracted epithelium (Fig. S1) and a unique furrow structure on the dorsal carapace (Fig. 3a). Based on these developmental features, we infer that the observed furrow represents the morphological state immediately preceding the onset of pumping behavior, formed by epithelial contraction before the new exoskeleton is fully expanded. Given the rarity of capturing this rapid transitional phase, this observation provides a valuable snapshot of the intermediate morphology. However, as this specimen failed to complete metamorphosis, we interpret this structure with caution, noting that artifacts associated with mortality cannot be fully excluded. The dorsal carapace of puerulus larvae was also observed, but the above furrow structure was not confirmed (Fig. 3a).

In insects, the folding and unfolding of the cuticle during metamorphosis has been the subject of study [3, 22]. However, such drastic morphological transformations are not unique to terrestrial arthropods. For example, Olesen reported that the branchiopod crustacean *Lynceus brachyurus* transforms from a dorsoventrally flattened larva to a laterally compressed juvenile [23]. This demonstrates that significant remodeling from a planar to a three-dimensional form occurs in aquatic crustaceans as well. Regarding the mechanism of deformation, it has also been proposed that a comparable mechanism may be present in small freshwater shrimp [24], thus suggesting that a similar phenomenon could occur during the transformation of phyllosoma to puerulus. While we acknowledge that the observed pumping movement is primarily essential for the withdrawal of the abdomen from the old exoskeleton, it is conceivable that the associated hydrostatic pressure also facilitates the unfolding of the cuticle surface, potentially utilizing such furrow structures, provided they represent the natural developmental state. Indeed, a previous study by Matsuda et al. (2017) confirmed that furrow extension contributes to the final shape [3]. Therefore, we first performed a computational simulation using three-dimensional mesh data from a specimen exhibiting the furrow structure. By flattening the dihedral angles, we demonstrated that furrow extension could indeed generate the observed carapace curvature (Fig. 3b). Conversely, this simulation also showed deformation in macroscopic structures that were not expected to extend, raising concerns about possible artifacts. Additionally, the computer simulations may use physical parameters that are unrealistic. To address these concerns, we conducted an analog simulation using a paper model. By reproducing the furrow pattern and constraining specific edges to mimic the boundary conditions of the original primordia (i.e., fixing the protruding upper segment and lateral margins), we found that the unfolding process successfully reproduced the global curvature (Fig. 3c). These results suggest that the characteristic furrows observed in a single specimen that died during the metamorphosis process may contribute to the final shape. Since phyllosoma is a planktonic organism that floats in the ocean, it is unclear how they achieve such a drastic deformation in the presence of the movement of the surrounding water. Additionally, puerulus larvae do not engage in feeding behavior for approximately 10 days until they metamorphose into the juvenile phase [12], which raises questions about the energy efficiency of the above transformation. In light of these intriguing questions, we hope that further analysis will be conducted.

### Consideration of how the furrow pattern forms

**Fig. 4:**
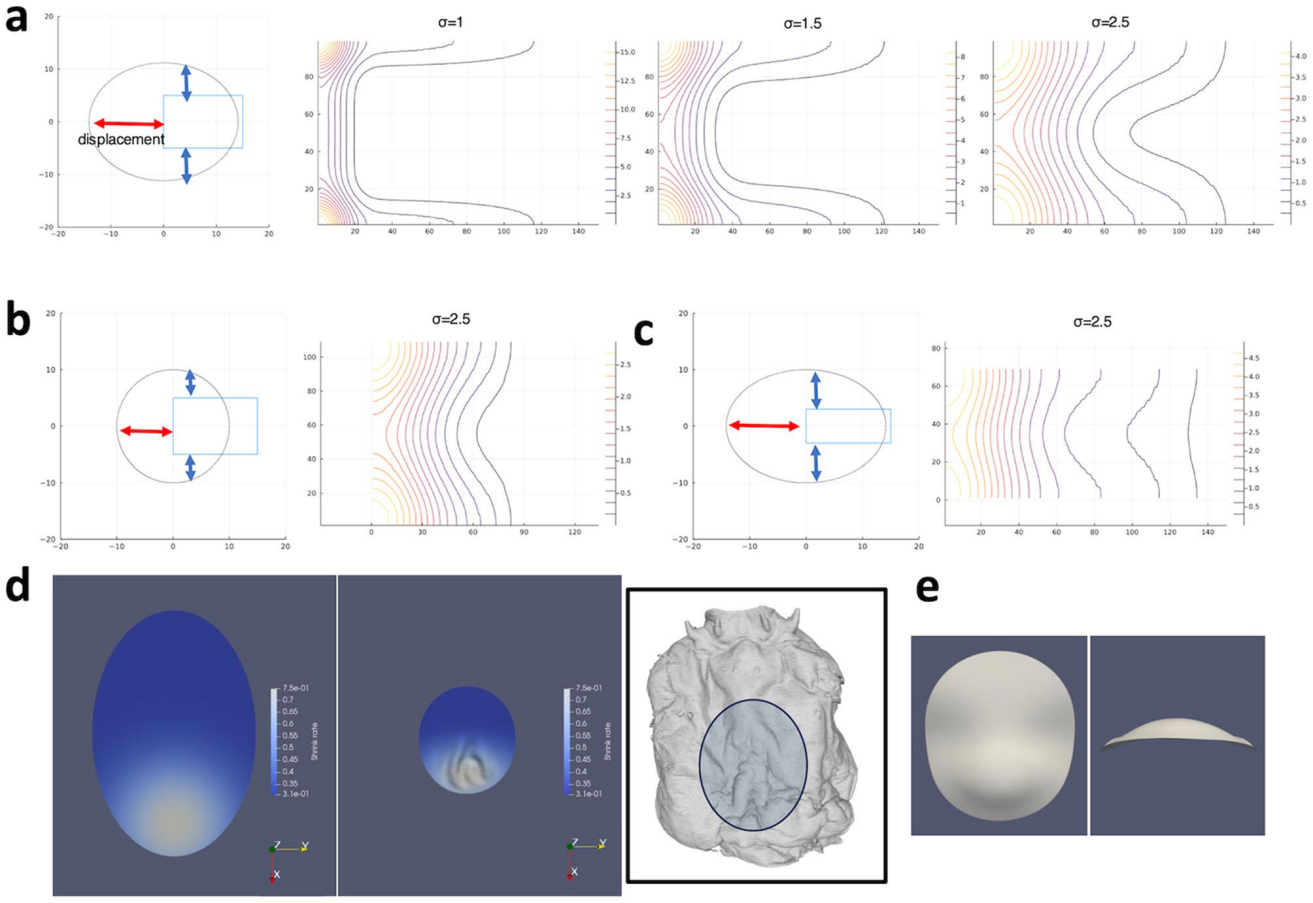
Reproduction of furrow patterns in two and three dimensions. (**a**) Two-dimensional (2D) simulation of displacement concentration pattern generated by approximating the phyllosoma and puerulus dorsal carapaces as an ellipse and a rectangle, respectively. (**b**) 2D pattern generated when the ellipticity is set to 1 (circular approximation). (**c**) 2D pattern generated when the rectangle is made narrower (increased aspect ratio). (**d**) Three-dimensional (3D) simulation of pre- and post-shrinkage morphologies, compared with the folding observed in the actual dorsal carapace primordium. (**e**) Unfolded projection of the 3D contracted morphology shown in (**d**)

Next, we consider how the distinctive furrow pattern, which is thought to contribute to the final morphogenesis, is formed. We noticed that in the aforementioned analog simulation with paper, the distinctive pattern was created by folding the paper with varying shrinkage rates in vertical and horizontal directions. While morphological transformation is fundamentally driven by cellular events such as proliferation, cytoskeletal reorganization, and tissue remodeling, these biological activities manifest macroscopically as mechanical forces, specifically, anisotropic tissue contraction. Therefore, we hypothesized that the physical buckling caused by these biologically driven contractions generates the furrow pattern. We first simplified the pre- and post-metamorphosis morphology, revealing differences in shrinkage rates in vertical and horizontal directions. By approximating the pre-metamorphosis shape as an ellipse and the post-metamorphosis shape as a rectangle (Fig. S2), we can model the transformation using a simple formula. The density distribution of the elliptical shape packed into the rectangle was considered, with the potential outcome of this configuration being the formation of the distinctive pattern. Initially, displacements of ellipses and rectangles were positioned on each side of the rectangle. For oblique sites that did not fit the rectangle, elements were placed at the vertices proportionally, based on vertical and horizontal excesses, and distributed along the edges following a Gaussian distribution. Subsequently, they were distributed inside the rectangle according to a Gaussian distribution in which the internal influence is varied by the variance parameter σ (Fig. S3). The density gradient pattern of the distributed values varied depending on the parameter σ of the Gaussian distribution (Fig. 4a). When σ is small, the pattern stops on the perimeter, and when σ is large, it penetrates more inside. By adjusting the parameters, we could create a pattern similar to that seen in the actual metamorphosis process (Fig. 4a). Assuming that buckling occurs at a certain threshold when density changes, it is suggested that the furrow pattern during metamorphosis may be caused by differences in shrinkage rates. Furthermore, the toy model created this time allows for alterations to the ellipticity of the ellipse and aspect ratio of the rectangle. An investigation was conducted to ascertain the impact of varying these parameters on the final pattern. For the σ, the value was selected to reproduce the similar pattern to the actual furrow observed during metamorphosis. As a consequence, it was determined that alterations to the ellipticity of the ellipse and aspect ratio of the rectangle resulted in corresponding changes to the final pattern (Fig. 4b, c). Of particular interest is the observation that as the disparity in shrinkage rates in vertical and horizontal directions decreases, the angle of the pattern becomes more gradual. In comparison to Palinuridae, the nisto larvae and adults of Scyllaridae have a lower curvature of the carapace and a more circular shape of the phyllosoma larvae [5]. This may be explained by our toy model, suggesting that further investigation into carapace changes in Scyllaridae phyllosoma larvae is warranted.

The aforementioned toy models focused solely on two-dimensional patterns. In practice, whether varying shrinkage rates at different locations allow folding in three dimensions remains unclear. A three-dimensional shrink model has been used to simulate the remodeling of beetle horns during the pupal-to-adult transition [25]. Following this approach, we employed a three-dimensional shrink model to examine whether folding occurs under anisotropic and gradient shrinkage, with greater contraction along the long axis. Anisotropic shrinkage was set so that the overall area decreased by a factor of 0.4, with an additional 2/3 reduction along the long axis compared to the short axis and the limitation of out-of-plane deformation was also imposed. The results demonstrated that anisotropic contraction with constrained out-of-plane deformation can form similar folding as observed in the Japanese spiny lobster metamorphosis process (Fig. 4d). Furthermore, within this model, we demonstrated that unfolding the fold (which corresponds to contraction without restricting out-of-plane deformation) can lead to curvature (Fig. 4e). These results support the idea that while the driving force is biological, the resulting morphology is governed by mechanical constraints, specifically, contraction under restricted out-of-plane deformation. Current knowledge about the limitation of out-of-plane deformation is limited, but it may be influenced by apical Extracellular Matrices (ECMs), such as cuticles. On the other hand, the folding of the carapace was observed in an individual that died during metamorphosis, and caution is required regarding whether this reflects the actual developmental phenomenon. At least, it is evident that the disparity in longitudinal and transverse contraction rates gives rise to the formation of a curved structure. However, understanding this drastic transformation requires considering not only surface mechanics but also anatomical reorganization. The significant decrease in body length observed during metamorphosis is likely a geometric consequence of redistributing tissue mass from a planar (phyllosoma) to a volumetric (puerulus) form. As the dorso-ventrally flattened body transforms into a three-dimensional structure, the anterior–posterior axis compresses, necessitating a spatial repacking of internal systems. Consequently, the arrangement of internal organs, including the digestive and nervous systems, must undergo substantial reorganization. For instance, the position of the mouth shifts to accommodate the new head geometry (Fig. 1). These internal anatomical changes imply that the reorganization is intrinsic and complex, extending beyond simple surface folding. While the present study has primarily described the deformation of the surface structure, we acknowledge that these internal structural reorganizations exert influence upon the surface dynamics. Nevertheless, it remains a fact that deformation can be described by considering the surface structure alone. In this study, the measurement of the mechanical parameters to be incorporated into the model proved technically challenging. Conducting experiments on the mechanics of crustaceans (e.g. nanoindentation) may refine the model in the future.

## Conclusions

In this study, our focus was on the deformation of the carapace, from that of the phyllosoma larvae to that of the puerulus larvae. First, using three-dimensional micro-computed tomography on fixed tissue, we accurately identified the deformation, including that of the carapace. Subsequently, the dynamics occurring in the carapace during the actual metamorphosis process were elucidated through live observation. Furthermore, scanning electron microscopy (SEM) observation of the carapace and carapace primordia of a single specimen that died during the metamorphosis process, in conjunction with digital-analog simulations, indicated that the distinctive furrow pattern on the cuticle surface may contribute to the final deformation. Furthermore, by approximating the deformation of the carapace from an ellipse to a rectangle, we demonstrated that a distinctive furrow pattern can be generated by the difference in the shrinkage rate in vertical and horizontal directions. Additionally, we found that various patterns emerge by deforming the ellipse and rectangle, suggesting potential applicability to other species. We also demonstrated that in three dimensions, local contractions can generate folds that curve upon unfolding. Collectively, these findings provide a biomechanical framework for understanding arthropod molting morphogenesis and offer new insights into the morphological evolution of Achelata.

## Supplementary Information

Below is the link to the electronic supplementary material.

Supplementary Material 1: Movie 1: Flat structure of the phyllosoma of *P. japonicus*. https://drive.google.com/file/d/1VJJu1Z4pD9e933MCt7-eu1PLysccvmmP/view?usp=drive_link

Supplementary Material 2: Movie 2 : Entire metamorphosis process from phyllosoma to puerulus of *P. japonicus*. https://drive.google.com/file/d/1cdP7yguSViCYV0bHS28trx4SpwGarv8g/view?usp=drive_link

Supplementary Material 3: Movie 3 : Carapace epithelium contraction during the early stage of metamorphosis from phyllosoma to puerulus of *P. japonicus*. https://drive.google.com/file/d/1H0f-dtDqXLo89XG3J4jJfrH7MtYjAi0n/view?usp=drive_link

Supplementary Material 4: Movie 4 : Pumping movement during the late stage of metamorphosis from phyllosoma to puerulus of *P. japonicus*. https://drive.google.com/file/d/1sC3g3lnTJe0C08wwD2H2K6ynxLx1_w0v/view?usp=drive_link

Supplementary Material 5

## Data Availability

The datasets used and/or analyzed during the current study are available from the corresponding author on reasonable request.
