# Supplementary material for: Morphogenesis of the carapace from phyllosoma to puerulus in the Japanese spiny lobster
Source: Zoological Lett. 2026 Apr 2;12:11. doi: 10.1186/s40851-026-00265-8 (PMC13339497; doi:10.1186/s40851-026-00265-8)
Supplement: Supplementary file 5 — Supplementary Material 5 [file 40851_2026_265_MOESM5_ESM.pdf]

Supplemental document

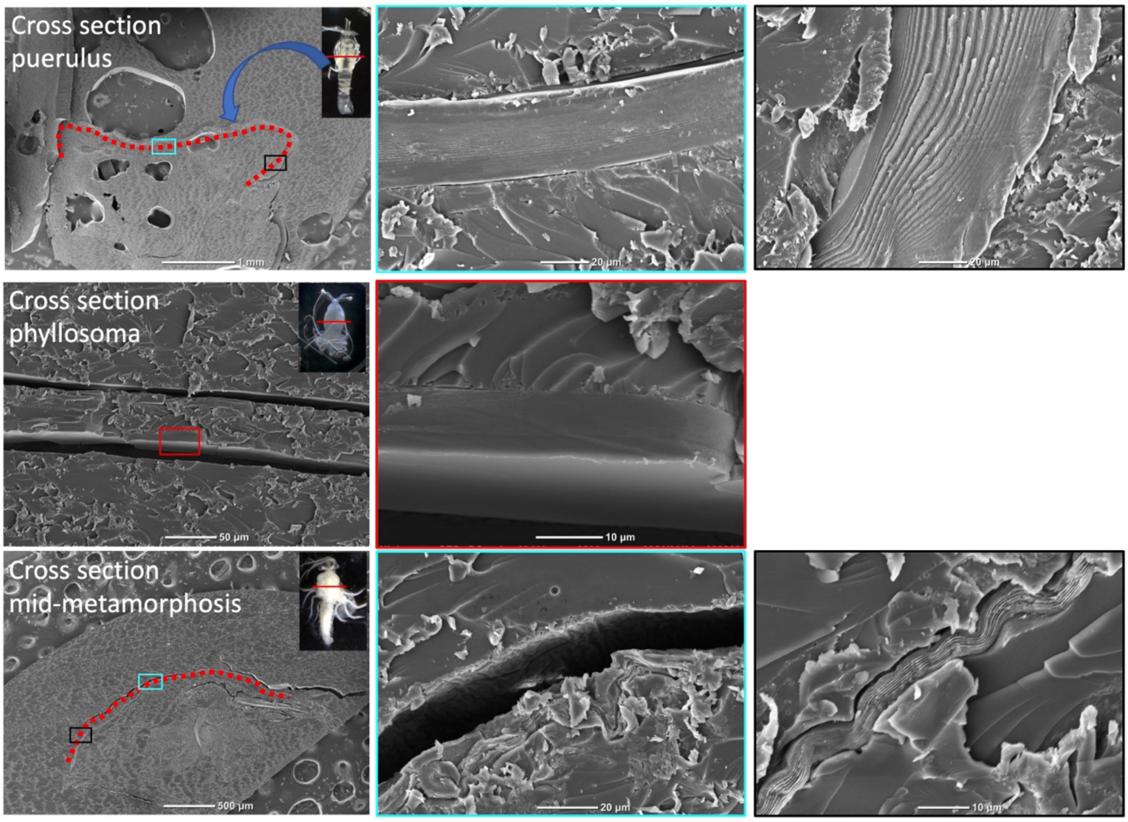

Fig. S1 Changes in cuticle structure during development

Fibrous structures were observed in the puerulus, whereas amorphous structures were seen in the phyllosoma, consistent with a previous study (10). In individuals transitioning from phyllosoma to puerulus (characterized by the presence of furrows), the underlying primordium, which was exposed by removing the outer phyllosoma cuticle, already exhibited a fibrous structure.

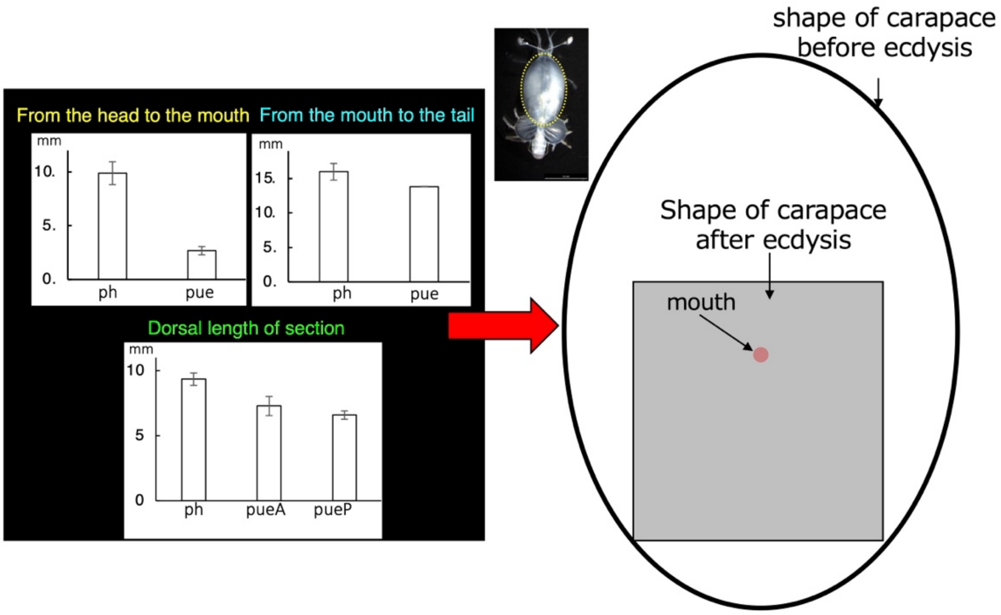

Fig. S2 Elliptical and rectangular approximations of the carapace of phyllosoma and puerulus.

The morphology of the phyllosoma and puerulus carapace was approximated as elliptical and rectangular, based on the morphological analysis in Fig. 1.

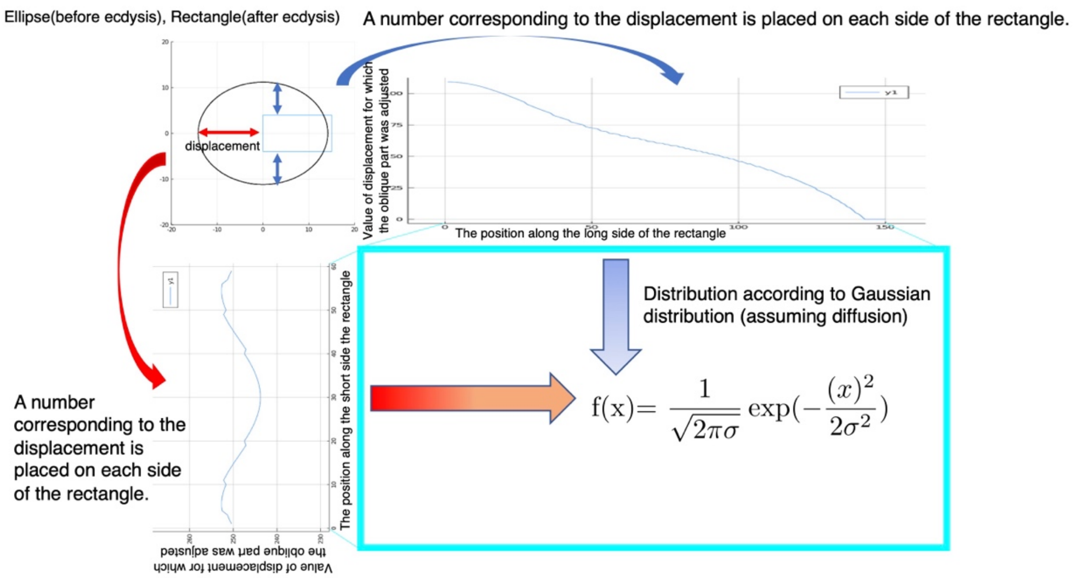

Fig. S3 Methods of the contraction thought experiment in two dimensions.

Rectangular and elliptical displacements were applied to each side of the rectangle and distributed within it according to a Gaussian distribution. For oblique sites that did not fit the rectangle, they were placed at the vertices proportionally, based on vertical and horizontal excesses, and distributed along the edges following a Gaussian distribution.
